# Disease prevalence and bacterial isolates associated with *Acropora palmata* in the Colombian Caribbean

**DOI:** 10.7717/peerj.16886

**Published:** 2024-02-28

**Authors:** Marco Garzon-Machado, Jorge Luna-Fontalvo, Rocio García-Urueña

**Affiliations:** Universidad del Magdalena, Santa Marta, Colombia

**Keywords:** Coral disease, Holobiont, Endangered species, Acropora palmata, Bacterial isolates

## Abstract

The decline in *Acropora palmata* populations in Colombian reefs has been mainly attributed to diseases outbreaks. The population size structure and prevalence of white pox and white band disease were evaluated in six localities of the Colombian Caribbean. Here, we aimed to isolate enteric bacteria and Vibrios from healthy and diseased coral mucus to relate its presence to the health status of *Acropora palmata*. The isolated bacteria were identified using molecular analyses with the 16S rRNA gene. Larger colonies had the highest percentage of the prevalence of both diseases. The strains that were identified as *Vibrio* sp. and *Bacillus* sp. were common in the healthy and diseased mucus of the holobiont. The *Exiguobacterium* sp. and *Cobetia* sp. strains isolated from diseased mucus may indicate maintenance and resilience mechanisms in the coral. *Enterococcus* sp. and other bacteria of the Enterobacteriaceae family were isolated from some localities, suggesting that probably contamination due to poor treatment of domestic wastewater and contributions from river discharges can affect coral health. The spatial heterogeneity of Colombian coral reefs exhibited variability in the bacteria, wherein environmental alterations can trigger signs of disease.

## Introduction

Coral reefs are considered one of the most biologically important ecosystems in the world, as they support a wide variety of hydrobiological resources (Moberg & Folke, 1999). In the last three decades, a drastic reduction in the percent living coral cover has compromised reef health (Hoegh-Guldberg, 1999; Gardner et al., 2003; Hoegh-Guldberg, Mumby & Hooten, 2007) and has been attributed to factors such as climate change, coastal development, overfishing, and the impacts of mass tourism practices (Buddemeier, Kleypas & Aronson, 2004).

An appropriate assessment of a coral population’s status should include estimations of the percent living coral cover, habitat type, environmental pressures, population size structure, recruitment dynamics, and health condition (Bruckner, 2011). In addition, characterizing impacts such as disease prevalence offers a broader view of the factors that interact with a population (Raymundo et al., 2008), which allows the identification of a susceptible population that can be affected by diseases or any other condition. These metrics offer a unique assessment over a specific temporal scale; for this reason, assumptions of population dynamics should be taken prudently and must be compiled with periodic assessments to provide reliable measures of population status over time.

The incidence and frequency of diseases are among the primary causes of habitat deterioration and loss in Caribbean reefs (Goreau et al., 1998; Green & Bruckner, 2000; Ainsworth et al., 2007). Populations of *Acropora palmata* and *A. cervicornis* that exhibite”-d high dominance in the past are now in decline due to the massive bleaching events that can be attributed to different factors including El Niño, a massive loss of herbivores (decline of parrotfish and sea urchins), and the appearance of specific diseases in Acroporids such as white band disease (WBD) and white pox (WPX) (Acropora Biological Review Team, 2005).

Corals have developed symbiotic interactions throughout their evolution due to their sessile life strategies, feeding mechanisms, metabolic sources, and physiological requirements (Blackall, Wilson & Van Oppen, 2015). Diverse groups of bacteria, viruses, and protozoa reside within the calcium carbonate coral skeleton, the soft internal tissues, and the surface mucus layer, which are involved in the health of the host (Rohwer et al., 2002; Blackall, Wilson & Van Oppen, 2015). The presence of certain bacterial groups and changes in bacterial communities because of environmental variations have repercussions for the emergence and outbreaks of disease in coral populations (Cróquer et al., 2013; Meyer et al., 2015). Establishing systematic microbial sampling during the manifestation of a disease is necessary due to their impact on coral health. Furthermore, some coral diseases have a relation between sewage pollution and the prevalence of disease, such as in black band disease (Jones et al., 2012), therefore study and isolate bacteria related to this kind of pollution in shallow waters is necessary to identify possible triggers of a disease.

The possible etiological agents of WBD include bacteria of the genera *Vibrio*, *Desulfovibrio*, and *Rickettsia,* in addition to members within the classes Alphaproteobacteria and Gammaproteobacteria (Pantos & Bythell, 2006). Two types of WBD (type I and II) (Klein & Vollmer 2011) have been described, which differ in their rates of daily progression, tissue loss associated between disease progression, and, in some cases, the possible pathogenic agent. However, there is a correlation with the presence of bacteria of the genus *Vibrio*, including *V. harveyi* and *V. carchariae* during disease progression in addition to a decrease in bacteria of the genus *Pseudomonas* (Bythell, Pantos & Richardson, 2004).

WPX is presumptively caused by the enterobacterium *Serratia marcescens*; this bacterium was isolated from diseased colonies of Florida reefs, and it was suggested to be the first case of a human gut bacterial species infecting a marine invertebrate (Patterson, Porter & Ritchie, 2002). Generally, the lesions are characterized by focal or multifocal areas of tissue loss distributed throughout the colony. However, other studies (Sutherland & Ritchie, 2004; Sutherland et al., 2010; Lesser & Jarett, 2014) did not find *S. marcescens* as the main trigger of WPX, suggesting that for a similar manifestation of this disease, more than one causative agent is possible.

The continental reef systems of the Colombian Caribbean are influenced by oceanographic events such as seasonal upwelling and unique geomorphological features that generate particular dynamics determined by thermal fluctuations, nutrient inputs, discharge from fluvial systems, and periodic high sedimentary loads (Restrepo & Kjerfve, 2000; Molares & Mestres, 2012; Andrade, 2015). Also, scarce information related to aspects of coral diseases and its microbiological agents within Colombian reefs are found and it is a lack of studies that should be addressed in this topic. The main literature that is available concerns about how the manifestation of a disease and the general view about them. This dynamic provides a suitable scenario to study disease incidence, defined in this study as the occurrence of both diseases (WPX and WBD), on the population structure and characterization of enteric and some non-enteric bacterial isolates of diseased and healthy colonies of *A. palmata* populations. Therefore, samples of mucus from healthy and diseased tissues were screened to isolate different strains, focusing on enteric bacteria, associated with WBD and WPX disease signs. Thus, this study allowed us to identify some enteric bacteria that reside in the coral mucus of *A. palmata* populations from Colombia that may be related to coral health through culture-dependent techniques.

## Materials & Methods

### Study area

**Figure 1:**
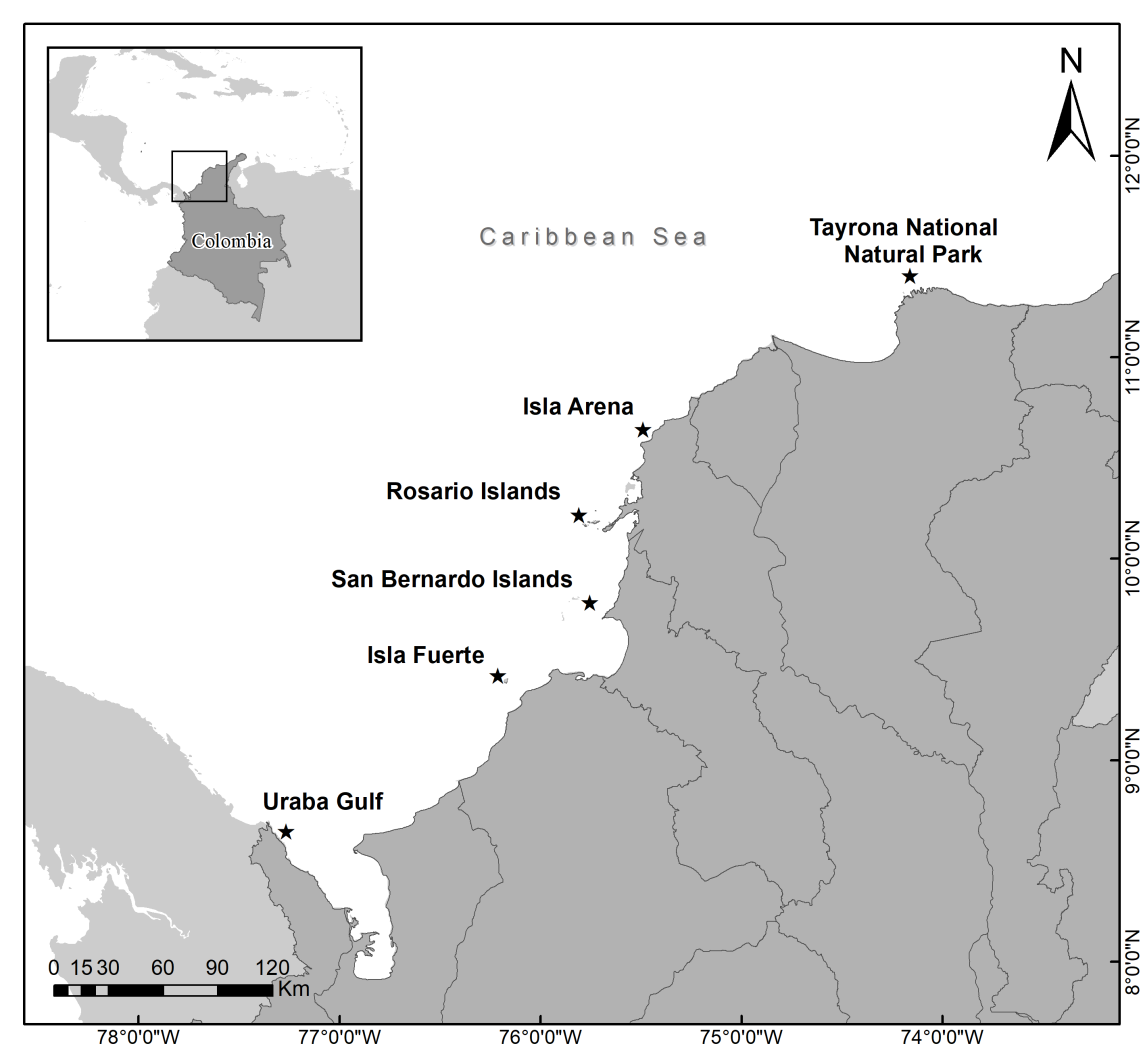
*Acropora palmata* populations in each locality surveyed along the Colombian Caribbean.

Six localities were selected and sampled based on their oceanographic influence in the Colombian Caribbean (Díaz et al., 2000): (i) Uraba gulf, where the reefs are influenced by discharge from the Atrato river and streams from Serrania del Darién, (ii) Isla Fuerte, where the reefs are grouped on shallow terraces that are influenced by the Sinú River, (iii) San Bernardo Islands, where the reefs have developed above the sedimentary bottoms, which are a byproduct of karstic depressions, (iv) Rosario Islands, where the reefs are in a dynamic interaction with the high sedimentary loads coming from el Canal del Dique (Alvarado et al., 1986), (v) Isla Arena, which is a diapiric islet that is under a strong influence from the sedimentary charges of the Magdalena River, and (vi) Tayrona National Natural Park (TNNP), which is characterized by the presence of seasonal upwelling systems; however, in their absence, the coral communities interact with river discharges from the Magdalena river and from the Cienaga Grande de Santa Marta (Díaz et al., 2000) (Fig. 1).

### Colony size structure and disease prevalence

During 2017 and 2018 once a year in each locality, *A. palmata* colonies were measured individually to construct the size class distribution calculating the area considering the maximum diameter (L) and the perpendicular diameter (l), assuming a ellipsoidal geometric shape of the colony followed by the equation: A = (L/2 * l/2) * *π* (Yap, Aliño & Gomez, 1992). At each colony, disease prevalence assessments were performed by analyzing photographic records and field notes taking from a diver approach. White-band disease was identified by decolored tissue at the base progressing to the tip of the colony, while White-pox was identified by irregular decolored spots on the colony tissue. Disease prevalence was obtained considering the number of colonies with each disease sign in the population divided by the total number of colonies in each locality. A population size structure histogram was constructed using the area data log_10_ transformed to obtain a good resolution for the small colonies and a normal distribution (Bak & Meesters, 1998). Importantly, except for the Tayrona locality, we could not establish an exact seasonal sampling in the other areas, therefore, the climatic influence was not accurately considered. Additionally, to test the null hypotheses that each disease was associated to the locality a chi-square analysis of contingency table was performed. Furthermore, a multidimensional contingency table was carried to test whether the disease occurrence, the locality and the size-class distribution were mutually independent, due to the results a partial independence contingency table was done to confirm if the combined disease occurrence was related to the size-class (Zar, 2010).

### Sample collection

At each location, through a roving approach mucus samples of two healthy colonies, two colonies with apparently WPX and two colonies with apparently WBD within the size class 4.29 cm2 log^10^-transformed, were collected from the coral surface using 5-mL sterilized syringes (needleless) (Barash et al., 2005; Gil-Agudelo et al., 2007; Koren & Rosenberg, 2008). Samples were transferred to 15-mL Falcon tubes and stored at 0–4 °C in darkness until laboratory processing. It is important to note that for Uraba gulf, Isla Fuerte and San Bernardo the transportation took more than three days until laboratory processing. The collected material was approved by the national authority “Parques Nacionales Naturales de Colombia with the entry permit 001 of March 6, 2017—PIC-DTCA 001-17”.

### Laboratory analysis

A volume of one mL of mucus was enriched in 10 mL of 0.1% peptone water adjusted to pH 9.0 (Donovan & Van Netten, 1995). Subsequently, the samples were incubated at 36 °C for 18 h, and 0.1 mL of the enriched solution was inoculated on glycerol marine agar and thiosulfate citrate bile sucrose agar (TCBS) (Lipp et al., 2002; Sampai et al., 2022). The cultures were incubated overnight at 36 °C, and each plate was cultured once more and incubated until the isolated colonies were pure (Patterson, Porter & Ritchie, 2002; Ritchie, 2006). It is pertinent to note that the selection of the temperature and culture media was tailored to isolate enteric bacteria, as it constituted the primary objective of the research.

### Bacterial identification

Bacterial isolates for molecular identification of the 16S gene were selected based on basic characteristics, such as Gram staining and bacterial morphology. Each isolate was grown overnight in broth for further DNA extraction. Extraction of DNA was performed with the MasterPureTM kit (EPICENTRE), and the universal primers for bacteria 27F (5′-AGAGTTTGATCMTGGATCAG-3′) and 518R (5′-ATTACCGCGGCTGCTGG-3′) were used to amplify the hypervariable V1–V3 region (Chakravorty et al., 2007). The PCR mixtures were as follows: 0.4 mM dNTP mix (10 mM dNTP mix; Promega, Madison, WI, USA), 1X reaction buffer (ABM, 10X PCR Buffer), 2 mM MgSO_4_ (ABM, 25 mM MgSO4), and 0.1 (U/mL) Taq Polymerase (MyTaq™ DNA Polymerase 50 (U/µL; Bioline, London, UK), each of which was completed to a volume of 25 µL. The PCR parameters used were 5 min at 95 °C; 1 min at 94 °C, 1 min at 63 °C, and 1 min at 72 °C for 35 cycles; 7 min at 72 °C; and 10 min at 4 °C. The amplified product was confirmed in 2% agarose and visualized in a UV chamber, these parameters were obtained after different trails were proven. The PCR products were sequenced in the forward direction using the dideoxy terminator cycle-sequencing method in an ABI 3730XL sequencer (Applied Biosystems, Foster City, CA, USA) at SSIGMOL, Universidad Nacional de Colombia, Bogotá, Colombia. DNA sequences were aligned and manually inspected for errors using BioEdit software (Hall, 1999). Bacterial identity was confirmed by free-access software BLAST, and accession numbers are given in Supplemental Information 1.

## Results

### Colony size structure and disease prevalence

**Figure 2:**
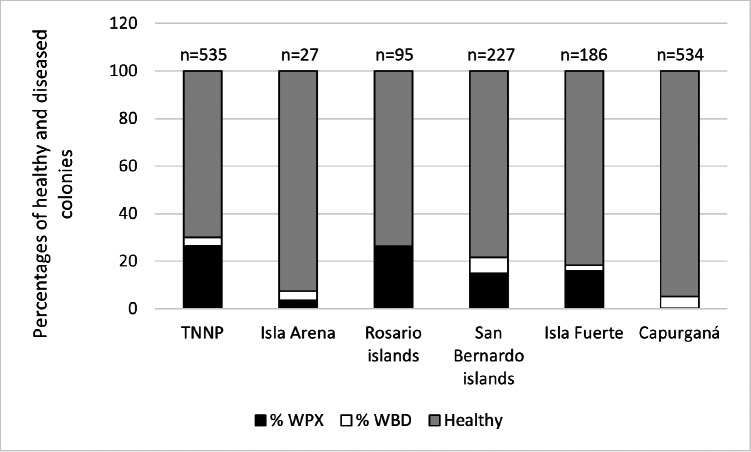
Disease prevalence and the proportion of healthy colonies among localities.

In general, a total of 1604 colonies from six localities were recorded by random sampling. WPX was more common than WBD, for WPX the disease was related to the locality (chi-square test, X${}_{\mathrm{0.05,3}}^{2}=11.07$; *P* = 167.7), on the contrary WBD was not related to the locality (chi-square test, X${}_{\mathrm{0.05,3}}^{2}$ = 11.07; *P* = 5.7), however, it is stated that this may be the result of the low occurrence of this disease. Overall, healthy colonies predominated, with an average of 81.85% among the colonies surveyed (Fig. 2). Isla Arena showed the higher percentage of healthy colonies (92.59%), the lowest values of healthy colonies were observed in the Rosario Islands (69.9%). Disease prevalence was dominated by WPX, which was highest in TNNP (26.54%), followed by the Rosario Islands (26.32%), and the other localities exhibited a disease prevalence of WPX below 20%. The prevalence of WBD was less than 10% in all localities (Fig. 2).

**Figure 3:**
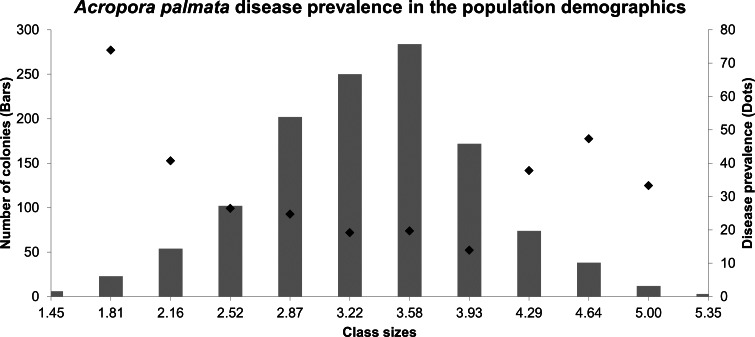
Size frequency distributions (bars) of Acropora palmata colonies in the Colombian continental reefs (2017). Disease prevalence is indicated by black circles.

The size-class distributions were grouped into 12 groups (1.45–5.71 cm^2^), most of which were represented by larger colonies between the sizes of 2.87–4.29 cm^2^ (74.42%), whereas the other classes were 25.5%. The highest prevalence of both diseases was found in colonies grouped in the second-class size which contains small colonies (73.91%), followed by large colonies within the tenth size class, colonies around 4.64–5.00 cm2 (47.36%). The lowest prevalence of both diseases combined was found in size class 6 (19.2%) (Fig. 3). The multidimensional contingency table showed that the disease occurrence, locality, and size-class were not mutually independent (chi-square test, X${}_{0.05,126}^{2}$ = 153.1; *P* = 421.05), concluding that the disease occurrence was present, no matter what class-size was evaluated (chi-square test, X${}_{0.05,1573}^{2}$ = 168; *P* = 154.9).

### Molecular analysis

Sixty-four pure bacterial isolates were obtained from healthy and diseased mucus samples, but only 33 were selected for molecular identification. The selection criteria were that at least one of each strain obtained from a diseased colony (if present) and one from a healthy colony were successfully purified from each locality. Some samples could not be sequenced due to poor-quality DNA. From the 32 sequenced strains, nine belonged to healthy coral, 12 to WBD, and 11 to WPX.

Thirteen strains were affiliated with *Vibrio* sp., six with *Bacillus* sp., three with *Enterococcus* sp., two with *Enterobacter* sp., and two with *Staphylococcus* sp. One strain was affiliated with *Salinicola* sp., *Exiguobacterium* sp., *Kocuria* sp., *Cobetia* sp., *Pseudomonas* sp., and *Pseudoalteromonas*. From healthy mucus, *Salinicola* sp. *Pseudoalteromonas* sp., *Enterobacter* sp., *Cobetia* sp., and *Kocuria* sp. were present in WBD samples, whereas *Exiguobacterium* sp. and *Pseudomonas* sp. in the WPX samples (Table 1).

**Table 1:** Bacterial isolates obtained from healthy and diseased colonies by molecular identification in the Colombian continental reefs. H, healthy; WPX, white-pox, and WBD, white band disease.

	**TNNP**			**Isla Arena**			**Rosario islands**			**San Bernardo islands**			**Isla Fuerte**			**Uraba Gulf**		
	**H**	**WPX**	**WBD**	**H**	**WPX**	**WBD**	**H**	**WPX**	**WBD**	**H**	**WPX**	**WBD**	**H**	**WPX**	**WBD**	**H**	**WPX**	**WBD**
*Kocuria oceani*			x															
*Bacillus endophyticus*								x										
*Bacillus flexus*																x		
*Bacillus megaterium*														x				x
*Bacillus pumillus*																x		
*Bacillus safensis*		x																
*Cobetia sp.*															x			
*Enterobacter sp.*						x												
*Enterococcus faecalis*											x	x						
*Escherichia coli*															x			
*Exiguobacterium profundum*														x				
*Pseudomonas aeruginosa*		x																
*Pseudoalteromonas sp.*																		x
*Salinicola sp.*													x					
*Staphylococcus*	x																	
*Staphylococcus epidermis*								x										
*Vibrio alginolyticus*		x																x
*Vibrio anguillarum*																x		
*Vibrio cincinnatiensis*																		x
*Vibrio neocaledonius*																		x
*Vibrio parahaemolyticus*			x							x								
*Vibrio sp.*	x			x				x						x				

The total number of bacterial isolates is shown in Table 1. However, *Vibrio* sp. were documented in each locality, followed by *Bacillus* sp. which were retrieved from three localities. Specifically, in TNNP, *Kocuria oceani* was found in WBD. In Isla Arena, *Vibrio* sp. were present in the healthy mucus, and *Enterobacter* sp. were present in the WBD samples. Rosario islands were represented by three strains obtained only from samples with WPX disease. Strains of *Enterococcus* sp. were exclusively isolated from San Bernardo Islands in disease mucus. Regarding Isla Fuerte, more diverse bacterial isolates were found, such as *Salinicola* sp. in healthy tissue; *Exiguobacterium* sp. from WPX samples, and *Cobetia* sp. and *E. coli* cultured from WBD samples. Finally, in Uraba Gulf, a strain of *Pseudoalteromonas* sp. was identified in WBD.

## Discussion

A decrease in *A. palmata* populations has been reported since the end of the 1970s, and this decline has caused a loss in the benthic habitat and physical complexity, and a decrease in reef diversity, resulting in impacts such as coastal erosion (Precht et al., 2002; Gardner et al., 2003). Although the diseases outbreaks such as the WPX and WBD in the Colombian continental Caribbean reefs compromise coral health, here we report that the proportion of healthy colonies at the time of sampling was higher, suggesting that *A. palmata* may have episodic recovery patterns given its ability to adapt and its resilience, however, to test this statement long-term surveys must be needed. Pearson (1981) proposed that the recovery rates of coral populations vary and depend on the type of impact (natural or anthropogenic) and suggested that the lack of apparent recovery may be a consequence of a drastic and permanent change in the environment (substrate).

When analyzing populations through size structures, demographic trends such as recruitment rates and the distribution of adult and juvenile individuals in a given space can be inferred (Soong, 1993; Meesters et al., 2001). In this study, larger sizes were present in all localities, which implies that populations may exhibit low recruitment rates even when the colonies have the appropriate sexual maturity size (Bak & Meesters, 1998). These results differ from those of González-Díaz et al. (2008) and Martínez & Gregorio-Quintal (2012), who found that Cuban and Venezuelan *A. palmata* populations tend to predominantly have sizes less than 10 cm, suggesting successful recruitment events that were favored by the biotic and abiotic components that operate in these reefs and facilitate the establishment of new individuals in a population.

Population studies of *A. palmata* in the Colombian Caribbean, such as those conducted by García-Urueña & Garzón-Machado (2020), determined that in the localities surveyed, populations can be healthy with a relatively good presence of small colonies, except for Isla Arena. Their data indicated that in all localities, significantly large colonies predominated. With the data presented here is noted how no matter the size of the colony surface it can present disease signs, future research should appoint if these lesions recover or can be detrimental to the colony and how this have implies on the reproductive output in the population.

The patch-type formation of *A. palmata* in TNNP is described as one of the most important formations in Colombian reefs; it is a continuous reef where colonies larger than 1,000 cm can be grouped together (García-Urueña, Garzón-Machado & Sierra-Escrigas, 2020). This may explain the high prevalence of WPX among the other sites according to the hypothesis that the proximity between healthy and diseased corals may have been subjected to infection through water-borne vectors, such as ballast water or un-treated domestic waters (Shore & Caldwell, 2019). In contrast, the disease prevalence in Uraba Gulf was one of the lowest; interestingly, these formations can be described as isolated colonies at least 4 m apart from each other (García-Urueña & Garzón-Machado, 2020), suggesting a successful sexual dispersion resulting in a noncontinuous population structure that contributes to limited infection *via* water-borne vectors (Rodriguez et al., 2008).

The disease prevalence for WBD was lower than that for WPX in each location, which may be important in terms of genetic disease resistance, as stated by Vollmer & Kline (2008) who found that three of 49 genotypes of staghorn coral *A. cervicornis* showed genetic resistance to WBD. As *A. palmata* shares a close evolutionary history, this species may exhibit some WBD genetic resistance in the Colombian reefs based on the data presented here. Although high densities of healthy colonies of *A. palmata* were surveyed, notably, the increase in the disease prevalence of WBD and WPX signs within the largest colonies could be the result of a high susceptibility and a decrease in metabolic resistance due to longevity by the time of the sampling (Mayor, Rogers & Hillis-Starr, 2006; Muller & Van Woesik, 2014). Therefore, research covering an extensive survey of population resistance to diseases, gene expression during disease, studies related to the designation of critical conservation areas coping with integral coastal management policies, and the identification of multiple environmental and biological associations with the presence of diseases should be prioritized for correct management of future predictions of disease outbreaks.

### Bacterial isolates

The metabolic features of the holobiont are entirely influenced by environmental conditions, and any anomalies such as nutrient inputs, temperature variation, sediment suspension, and other anthropogenic tensors have negative impacts on coral health, resulting in the colonization of pathogenic and opportunistic bacteria or dysbiosis of the bacterial groups residing in the coral (Sunagawa et al., 2009). In particular, each locality in the surveyed Colombian reefs has its own oceanographic aspects that can affect the balance of coral health by changing the bacterial composition or the colonization of pathogens that have repercussions on coral diseases, which can be associated with the probiotic coral hypothesis (Reshef et al., 2006), which suggests that there is a dynamic relationship between the different symbiont microorganisms and the environmental conditions that interact with the holobiont. Next generation sequencing studies must be done to clarify support this statement.

Most of the bacterial isolates in this study belong to Enterobacteriaceae, which is considered a highly pathogenic family among biological systems (Brenner & Farmer, 2015). In marine organisms, its incidence and affectation trigger disease events in groups such as mollusks, fishes, and marine mammals (Guo & Ford, 2016). Although Enterobacteriaceae are a common member in different coral tissues, its role is unclear, but some data suggest that this family disrupts the holobiont wellbeing (Morrow et al., 2012; Roder et al., 2014). For example, Leite et al. (2018) found a relationship between the dissolved nitrogen concentration and the abundance of members of the family Enterobacteriaceae within different Brazilian coral populations, and they found a significant positive correlation for disease presence and polluted urban focuses. This finding indicates that this group may work as a proxy to establish control and conservation strategies against epizootic events in corals located near urban areas.

Uraba Gulf was characterized by the low presence of WBD. This locality receives contributions from the Atrato river characterized by the high abundance of total organic carbon and total nitrogen (Velez-Agudelo & Agudelo-Ramirez, 2016), and although WBD is not strictly related to river discharges, the presence of extra nutrients can favor the overgrowth and colonization of pathogenic bacteria that express virulence factors (Pollock et al., 2011). The causal agent for this disease is uncertain; however, a direct relationship has been found with representatives of the genus *Vibrio* sp. in different tissue isolations from diseased corals (Gil-Agudelo et al., 2007; Gignoux-Wolfsohn & Vollmer, 2015). Three strains of *Vibrio* sp. corresponded to WBD, which coincides with other studies, this also validated that the presence of *Vibrio* sp. may play a critical role in holobiont maintenance during the healthy and diseased state.

Many of the isolated and sequenced strains of the bacteria in this study coincide with those of a previous report (Sweet, Croquer & Bythell, 2014) in which bacteria of the genus *Vibrio* sp. and *Bacillus* sp. were isolated from healthy and diseased colonies of *A. cervicornis*, suggesting that there are bacterial groups that are closely linked to the appearence of diseases in acroporid corals. Pantos & Bythell (2006) used independent culture techniques in colonies of *A. palmata* with WBD and found a significant proportion of representatives of the Gammaproteobacteria class. In the present study, the sequenced bacteria were grouped primarily within this taxonomic level, indicating that members of Gammaproteobacteria may be a common group involved in advancement of the disease.

*Exiguobacterium* sp. were isolated from a colony with WPX in Isla Fuerte, representing a strain of interest, as it is part of the healthy microbiome in *A. palmata* and participates in a beneficial manner within the host; this strain retards the growth of pathogenic bacterial populations by inhibiting glycosylated compounds, such as those present in *S. marcescens,* presumably the causative agent of WPX (Krediet et al., 2013). The presence of this strain suggests that during the emergence of disease signs, some beneficial bacteria participate in the equilibrium and health of the host. *Cobetia* sp. were also observed in this locality, which participate in habitual metabolic processes such as the assimilation of carbohydrates, amino acids and other metabolic processes (Badhai, Ghosh & Das, 2016). Although both were isolated from diseased mucus, the strains are probably present because of a gradual change in the composition of bacteria during progression of the disease (Cárdenas et al., 2012; Roder et al., 2014). In *A. palmata*, this finding may suggest that the species harbors beneficial microbes with the potential to combat and stop advancement of the disease if environmental conditions are favorable but as the data presented here is preliminary in terms of microbiota, long-term studies combined with current methodologies should address this statement.

*Kocuria* sp. was isolated in TNNP, where the species were shown to be representative of the pathogenic microbiome that initiates signs of disease when combined with other bacteria (Sweet & Bulling, 2017). *Enterobacter* sp. were isolated in Isla Arena from WBD mucus. This pathogen and its representatives within the family Enterobacteriaceae contribute to the advancement of different diseases, such as black band and white plague; however, they have also been found in low proportions in healthy mucus (Frías-lópez et al., 2002; Morrow et al., 2012; Roder et al., 2014). The presence of *Enterobacter* sp. should be revised in future studies considering how the periodic contributions from the Magdalena River may affect the coral health. On the San Bernardo Islands, two strains of *Enterococcus* sp. were recovered from diseased mucus, and although their presence cannot be attributed to the emergence of disease, they appear to be associated with waters contaminated by surrounding human populations. Therefore, bacteria of fecal origins may contribute to and influence diseases in *A. palmata* (Sutherland et al., 2010).

## Conclusions

This research aimed to survey most of the *A. palmata* population sites along continental Colombian reefs. The findings showed an important presence of healthy colonies, but they are highly susceptible to partial mortality due to diseases, primarily WPX. Little is known about their specific recovery rates and the severity of these epizootic events in Colombian populations. Research covering this area must be performed in the future to properly design management plans for this species. Populations present in TNNP, Uraba Gulf, and San Bernardo islands should be addressed as priority sites where policies and management plans must be implemented to protect this delicate population.

Data presented here provide a general description of the presence of some of the enterobacteria isolated in *A. palmata*. The manifestation of a particular disease may be due to a change in the composition of the bacteria and not to the colonization and infection of a pathogen from the environment. In a general sense, one of the pathogenic bacterial isolates (*Vibrio* sp.) was retrieved from healthy mucus, which suggest that *Vibrio* sp. may be a common resident during this state, but it should be explored whether it expresses pathogenicity depending on its abundances within *Acropora palmata*. Other isolates with reported beneficial roles were obtained from diseased colonies (*Exiguobacterium* sp. and *Cobetia* sp.). Its presence may raise questions about how *Acropora palmata* colonies may resist to maintain its normal functions during disease manifestation, and further metabolomic research should be carried to assess these statements. Thus, compositional changes are determined to be the primary cause of the metabolic imbalance that affects coral health, that in some cases are triggered by environmental variations that, in this case, may be influenced by the river discharges affecting each locality. Future analyses that relate environmental proxies with coral health should be consider in the country to propose specific management strategies depending on the locality. Notably, coral diseases are the result of complex interactions between macro- and microorganisms with environmental variables, and therefore, the results here must be analyzed as preliminary.

## Supplemental Information

10.7717/peerj.16886/supp-1 Supplemental Information 1 Accession numbers of the bacterial isolates

10.7717/peerj.16886/supp-2 Data S1 Raw data from disease prevalence and size frequency distribution

## Additional Information and Declarations

### Field Study Permissions

The following information was supplied relating to field study approvals (i.e., approving body and any reference numbers): The collected material was approved by the national authority “Parques Naturales Nacionales de Colombia” (001 of March 6, 2017–PIC-DTCA 001-17)

### DNA Deposition

The following information was supplied regarding the deposition of DNA sequences: The bacterial isolates described are available at GenBank:
MK932872.1, MK933266.1, MK933267.1, MK933281.1, MK933764.1, MK933765.1, MK933772.1, MK933778.1, MK933824.1, MK934135.1, MK934174.1, MK935341.1, MK935558.1, MK935570.1, MK935696.1, MK935697.1, MK935698.1, MK935699.1, MK935700.1, MK935702.1, MK935704.1, MK935705.1, MK935707.1, MK936044.1, MK936046.1.

### Data Availability

The following information was supplied regarding data availability: The raw data are available in the Supplemental Files.
